# The *37TrillionCells* initiative for improving global healthcare via cell-based interception and precision medicine: focus on neurodegenerative diseases

**DOI:** 10.1186/s13041-024-01088-4

**Published:** 2024-04-11

**Authors:** Benoit Coulombe, Thomas M. Durcan, Geneviève Bernard, Asmae Moursli, Christian Poitras, Denis Faubert, Maxime Pinard

**Affiliations:** 1 Translational Proteomics Laboratory, Institut de Recherches Cliniques de Montréal, Montreal, QC H2W 1R7 Canada; 2 Department of Biochemistry and Molecular Medicine, Université de Montréal, Montreal, QC Canada; 3 The Neuro’s Early Drug Discovery Unit (EDDU), McGill University, Montreal, Canada; 4 Departments of Neurology and Neurosurgery, Pediatrics and Human Genetics, McGill University, Montreal, Canada; 5 Department Specialized Medicine, Division of Medical Genetics, McGill University Health Centre, Montreal, Canada; 6 Child Health and Human Development Program, Research Institute of the McGill University Health Centre, Montreal, Canada; 7 Mass Spectrometry and Proteomics Platform, Institut de Recherches Cliniques de Montréal, Montreal, QC H2W1R7 Canada

**Keywords:** 37TrillionCells, Single-cell proteomics, SCoPE2-MS, Cell-based interception and precision medicine, iPSC, Organoid, Leukodystrophy, POLR3-HLD

## Abstract

One of the main burdens in the treatment of diseases is imputable to the delay between the appearance of molecular dysfunctions in the first affected disease cells and their presence in sufficient number for detection in specific tissues or organs. This delay obviously plays in favor of disease progression to an extent that makes efficient treatments difficult, as they arrive too late. The development of a novel medical strategy, termed cell-based interception and precision medicine, seeks to identify dysfunctional cells early, when tissue damages are not apparent and symptoms not yet present, and develop therapies to treat diseases early. Central to this strategy is the use of single-cell technologies that allow detection of molecular changes in cells at the time of phenotypical bifurcation from health to disease. In this article we describe a general procedure to support such an approach applied to neurodegenerative disorders. This procedure combines four components directed towards highly complementary objectives: 1) a high-performance single-cell proteomics (SCP) method (*Detect*), 2) the development of disease experimental cell models and predictive computational models of cell trajectories (*Understand*), 3) the discovery of specific targets and personalized therapies (*Cure*), and 4) the creation of a community of collaborating laboratories to accelerate the development of this novel medical paradigm (*Collaborate*). A global initiative named *37TrillionCells* (37TC) was launched to advance the development of cell-based interception and precision medicine.

## *37TrillionCells*, a collaborative research initiative for the development of cell-based interception and precision medicine

**Fig. 1:**
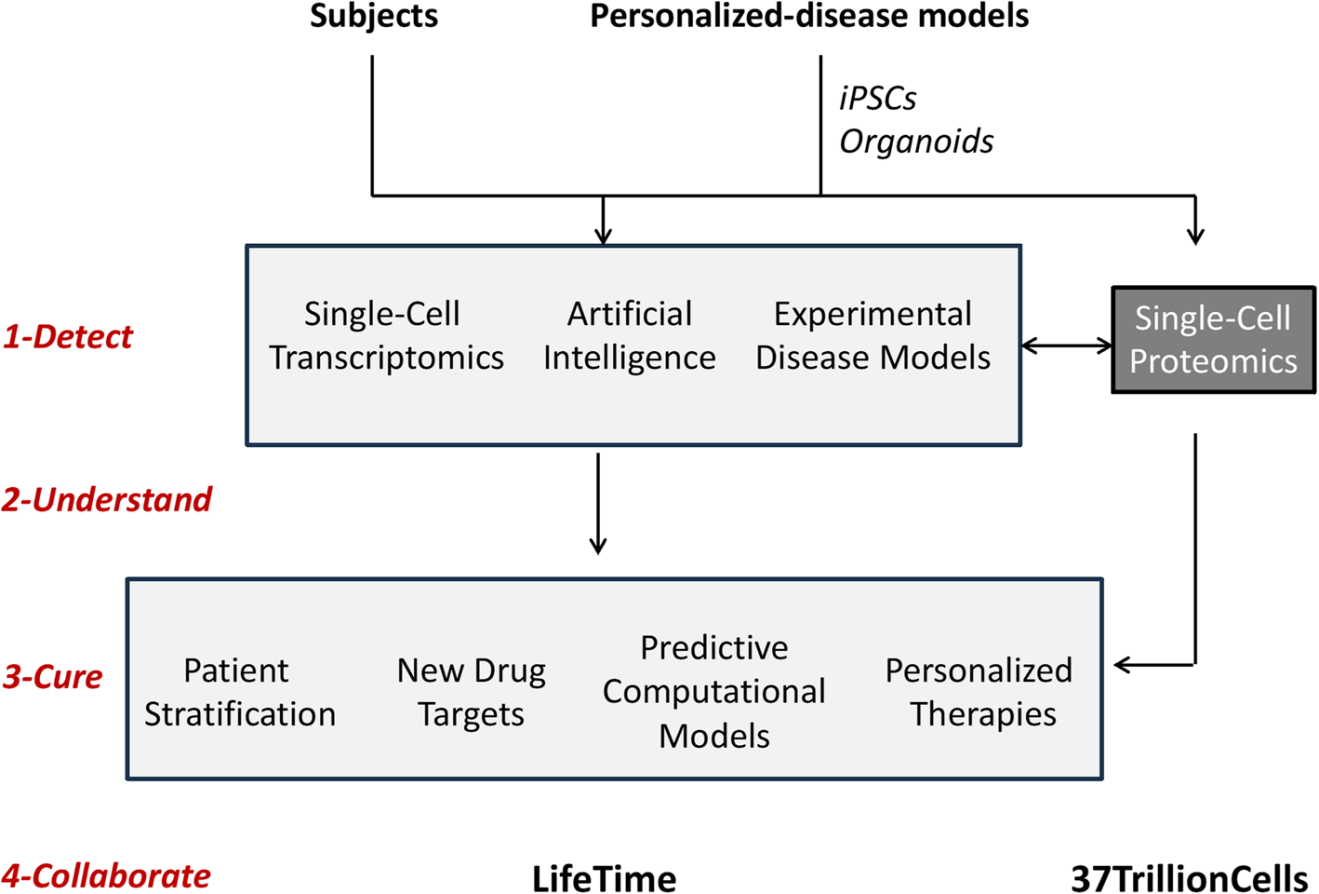
Overview of the LifeTime and 37TrillionCells initiatives

In 2020, the concept of “cell-based interceptive medicine” emerged in Europe through a novel biomedical research initiative called LifeTime [1], led by the Max-Delbrück-Center for Molecular Medicine and the Charité in Berlin and the Curie Institute in Paris [2]. To complement LifeTime, a Canadian initiative named *37TrilllionCells* (37TC) [3] was launched to add single-cell proteomics to the various technologies already included in the LifeTime program. 37TC is led by four institutions in the province of Québec (Canada): the University of Montreal, the research institute of the McGill University Health Centre (MUHC), the Montreal Neurological Institute and Hospital (MNIH), and the Montreal Clinical Research Institute (IRCM), and is open to new participating centres. Figure 1 schematizes the various components of LifeTime and 37TC. Although LifeTime is directed towards most human organs and several associated diseases, 37TC focuses on the brain and neurodegenerative diseases, mainly but not limited to leukodystrophies. In future development, 37TC plans to integrate other neurodegenerative diseases in its pipeline, including Alzheimer’s disease, amyotrophic lateral sclerosis and others.

The overall goal of 37TC is to implement and operate a novel multidisciplinary, muti-institutional technology consortium for the development of cell-based interception medicine, where researchers are developing and characterizing a series of induced pluripotent stem cells (iPSCs), iPSC derived cells and organoid models recapitulating the development of normal brain cells as well as diseases specifically affecting the white matter of the nervous system such as leukodystrophies and related conditions [4–6]. High performance single-cell proteomics (SCP) is used to profile the proteome of each cell type at a single-cell resolution in order to follow specific gene expression programs in detail and to identify targets for the discovery of drugs that can reprogram gene expression and help to cure diseases. These therapeutic drugs could target various cellular molecules including epigenetic or transcription factor [7, 8].

Leukodystrophies are severe illnesses that predominantly impact children, causing a gradual decline in abilities and often resulting in early mortality, occurring within months to a few years after the disease begins. These conditions specifically target the brain's white matter or myelin and stem from abnormalities either in myelin formation (hypomyelinating) or disruptions in myelin maintenance (non-hypomyelinating) [9]. There are over 50 different leukodystrophies/genetically determined leukoencephalopathies. To this day, our group has discovered eight leukodystrophy genes, including four genes causing POLR3-related leukodystrophy (POLR3-HLD), one of the most common hypomyelinating leukodystrophy. Indeed, POLR3-HLD or 4H leukodystrophy (Hypomyelination, Hypodontia and Hypogonadotropic Hypogonadism) is caused by biallelic pathogenic variants in five genes encoding subunits of the ubiquitous enzyme RNA polymerase III (Pol III), four of which were discovered by our group [10–13]. Even if it was shown that most of these mutations cause Pol III assembly defects, how they lead to hypomyelination is poorly understood. Similarly, the physiopathology of many leukodystrophies, including disorders caused by mutations of genes coding for tRNA synthetases (e.g., *EPRS1* [14], *VARS1* [15]) is also poorly understood.

Considering that new leukodystrophy genes are discovered nearly each year, there is a clear need for novel approaches to tackle the causative mechanisms and we posit that SCP is a strong candidate. A new high-performance SCP platform has been established and is made available to the research community as a fee-for-service facility via 37TC, contributing to the recruitment of new scientists and the training of highly qualified personnel. 37TC, in addition to an iPSC and organoid specialized preparation module, is seeking to develop sufficient capacity to support the community, as this technology becomes transformative in biomedical research for the years to come.

## Cell-based interception and precision medicine: A new medical paradigm for early detection, stratification and treatment of diseases

For each human being, one cell with one genome, the egg, produces about 37 trillion cells, with more than 1000 different cell types [16]. The genome is read differentially over space and time to drive cells towards their destinies, to fulfil specific roles in the formation and homeostasis of each tissue. Perturbation of these “normal” phenotypic cell trajectories are at the origin of diseases. Our ability to profile global gene expression at single-cell resolution provides a new rationale to track and understand how, when, and why cells bifurcate away from their normal trajectories. Reconstitution of disease progression is crucial to design interventions that can intercept and correct aberrant trajectories or states before it is too late, and pathologies escape treatment. As mentioned above, this strategy is part of an emerging medical paradigm, cell-based interceptive medicine [2]. Because the onset of several rare neurodegenerative diseases, including leukodystrophies, takes place during childhood, they often have a high cost and impact for society, especially affecting quality of life of children and their families.

Cell-based interception and precision medicine can be divided into four main components/steps: *Detect, Understand, Cure, Collaborate*.

1. *Detect*. The Detect component is aimed at identifying disease cells early according to their protein expression profiles at single-cell resolution. A high-throughput, high-sensitivity SCP workflow for profiling the proteome of each cell of tissues (or related models) over time has been implemented. The data is used to build computational models to serve as early biomarkers for neurodegenerative disorders. For early development of the Detect component, single-cell proteomics by mass spectrometry (SCoPE2-MS) published in 2021 by the laboratory of Prof. Nikolai Slavov at Northeastern University, USA, with a Thermo Q-Exactive instrument [17], is used. This method uses multiplexing with tandem mass tag (TMT) reagents. Since SCoPE2-MS development, novel instrumentation has been developed that greatly improves throughput (i.e., number of samples analyzed per day) and sensitivity (i.e., number of proteins identified per cell). As an improvement of this component, we are aiming to combine the use of the Bruker tims TOF SCP/Ultra mass spectrometer and the Cellenion cellenONE single-cell preparation robot. This combination of technologies will increase the SCP platform performance manyfold.

SCP is a rapidly evolving technology in which the proteins are analyzed on a cell-by-cell basis. Because the protein content of one cell is small, special experimental conditions must be applied to detect larger number of proteins. Detergents should be avoided and, the pipetting and transfer from one tube to another must be minimized while including a mixture of peptides to reduce plasticware absorption [18, 19]. Cells can be sorted by fluorescence-activated cell sorting (FACS) using specific antibodies and the various cell populations can be tagged with specific TMT molecules as a chemical barcode to increase the number of proteins per analysis, thereby increasing sensitivity and throughput.

The Detect component will identify proteins and protein complexes/networks that are specifically expressed in each iPSCs and organoids derived from patients, providing tools to identify diseased cells and helping to understand mechanisms of brain development in both healthy and disease conditions (see below).

2) *Understand.* The Understand component is aimed at developing experimental models (iPSCs and organoids) enabling studies of cell trajectories during normal brain development and brain diseases such as leukodystrophies. In preliminary experiments, the iPSC line AIW002-02 is used to produce a set of neuronal cell types, including dopaminergic neural precursor cells (DA-NPC) [20], neurons (DA neurons), oligodendrocyte precursor cells (OPCs), oligodendrocytes [21] and astrocytes [22]. The AIW002-02 line was previously reprogrammed from peripheral blood mononuclear cells. Other iPSC lines have also been made and are readily available to ensure that both male and female are represented equally [23]. After differentiation, cells are dissociated and processed for flow cytometry (FC), also as described by Thomas et al. [24]. Isolated cells are used for proteomics analysis. Midbrain organoids will be derived from iPSCs and dissociated after differentiation [25]. Cells will then be processed for FC and analyzed by proteomics. In these experiments, FC uses a panel of 13 antibodies that provides expression levels of the various proteins used to cluster the cells. A number of protein factors that are identified as being regulated during cell differentiation or environment response are characterized using classical molecular biology methods. Affinity purification coupled with MS (AP-MS) [11, 13, 26, 27], proximity biotinylation (BioID) [28] and AI-driven software such as PAIRS [29] are used to identify interactors. siRNA silencing or CRISPR/Cas9 knockouts serve to assess their roles in differentiation.

Changes in single-cell proteome profiles from various iPSC lines derived from patients and during their maturation into organoids will generate clues on mechanisms of disease progression and the proteins involved in the process. Dysregulated protein expression profiles will be used to screen for bioactive molecules able to correct protein expression (see below).

3) *Cure.* The Cure component is aimed at selecting protein factors differentially expressed during disease model evolution and assess them as targets for the discovery of drugs that can restore normal gene expression programs. A similar procedure was used to repurpose the FDA-approved drug Riluzole as modulating Pol III assembly in the presence of a leukodystrophy-causing subunit variant [27]. Small-molecule inhibitors or activator, target-specific antibodies or inhibitory RNAs are three classes of bioactive compounds that are privileged for this module. We posit that restoring normal gene expression programs may have the ability to counter disease progression.
4) *Collaborate.* The Collaborate component is a core service made available on a fee-for-service basis to the community. This module will accelerate the development of cell-based interception medicine and the participation of laboratories and centers worldwide to the evolution of this new medical paradigm.

37TC deploys and strengthens the research infrastructure necessary to develop new technologies dealing with SCP, innovative 3D tissue experimental models, and artificial intelligence. It will identify the key cellular trajectories during brain development and those at stake in brain diseases. In combination with other LifeTime components such as nuclear dynamics and chromatin organization data, the resulting technological advances and scientific discoveries function as a lever for future breakthroughs and a new way to address many diseases beyond those targeted here with a long-term impact on society, medicine and the economy.

## The promises of single-cell analysis

Over the past years, single-cell transcriptomics (also named sc-RNAseq) has been invaluable not only to study the progression of different diseases, but also to study the biology of various tissues and organs [30]. Since proteins are the functional actors of cells and because RNA levels often do not correlate with protein levels, we think that SCP is a method of choice to profile cells in any given tissue. Proteomic studies are likely to provide details much further beyond sc-RNAseq, which can include posttranslational modifications (PTMs). For example, phosphorylation profiles of peptides can be differentially detected in different cells. Researchers have identified more than 400 different types of PTMs that regulate the function of proteins [31]. We expect to establish the roles for some of them during brain cell differentiation and/or disease progression. In addition, the stoichiometry of protein components that are part of given complexes is likely to define whether whole complexes or rather individual subunits are regulated during cell differentiation or disease progression. For example, if Pol III (or any other multisubunit complexes) is regulated during differentiation of a given cell type, we expect to find a difference in expression of multiple polymerase subunits and/or their interactors in our analysis [32]. These previous points are in addition to changes in protein expression levels occurring during normal or disease cell evolution. In the case of POLR3-HLD, we trust that changes in the proteome during disease progression will illuminate causative mechanisms and reveal the link between Pol III defects and aberrant myelin metabolism. 

## Data Availability

Not applicable.

## Abbreviations

37TC: *37TrillionCells* Initiative
AP-MS: Affinty purification coupled to mass spectrometry
Pol III: ARN Polymerase III
AI: Artificial intelligence
DA-NPC: Dopaminergic neural precursor cells
FC: Flow cytometry
FACS: Fluorescence-activated cell sorting
FDA: Food and drug administration
iPSCs: Induced pluripotent stem cells
DA neurons: Neurons
OPCs: Oligodendrocyte progenitor cells
POLR3-HLD: POLR3-related leukodystrophy
PTM: Posttranslational modifications
BioID: Proximity biotinylation
RNA: Ribonucleic acid
SCP: Single-cell proteomics
sc-RNAseq: Single-cell RNA sequencing
siRNA: Small interfering RNA
TMT: Tandem mass tags
